# HDX and Native Mass Spectrometry Reveals the Different Structural Basis for Interaction of the Staphylococcal Pathogenicity Island Repressor Stl with Dimeric and Trimeric Phage dUTPases

**DOI:** 10.3390/biom9090488

**Published:** 2019-09-14

**Authors:** Kinga Nyíri, Matthew J. Harris, Judit Matejka, Olivér Ozohanics, Károly Vékey, Antoni J. Borysik, Beáta G. Vértessy

**Affiliations:** 1 Department of Applied Biotechnology and Food Sciences, Budapest University of Technology and Economics, 1111 Budapest, Hungary; 2 Institute of Enzymology, Research Centre for Natural Sciences, Hungarian Academy of Sciences, 1117 Budapest, Hungary; 3 Department of Chemistry, King’s College London, Britannia House, London SE1 1DB, UK; 4 Institute of Organic Chemistry, Research Centre for Natural Sciences, Hungarian Academy of Sciences, 1117 Budapest, Hungary; 5 Department of Medical Biochemistry, Semmelweis University, 1085 Budapest, Hungary

**Keywords:** dUTPase, inhibition, interaction surface, Stl staphylococcal repressor

## Abstract

The dUTPase enzyme family plays an essential role in maintaining the genome integrity and are represented by two distinct classes of proteins; the β-pleated homotrimeric and the all-α homodimeric dUTPases. Representatives of both trimeric and dimeric dUTPases are encoded by *Staphylococcus aureus* phage genomes and have been shown to interact with the Stl repressor protein of *S. aureus* pathogenicity island SaPIbov1. In the present work we set out to characterize the interactions between these proteins based on a range of biochemical and biophysical methods and shed light on the binding mechanism of the dimeric φNM1 phage dUTPase and Stl. Using hydrogen deuterium exchange mass spectrometry, we also characterize the protein regions involved in the dUTPase:Stl interactions. Based on these results we provide reasonable explanation for the enzyme inhibitory effect of Stl observed in both types of complexes. Our experiments reveal that Stl employs different peptide segments and stoichiometry for the two different phage dUTPases which allows us to propose a functional plasticity of Stl. The malleable character of Stl serves as a basis for the inhibition of both dimeric and trimeric dUTPases.

## 1. Introduction

Infections caused by *Staphylococcus aureus* are hazardous for both humans and livestock especially since *S. aureus* strains develop resistance and adapt to the new hosts rapidly via horizontal gene transfer (HGT)[1,2]. Highly mobile *S. aureus* pathogenicity islands (SaPI) play a key role in this process since they frequently carry genes encoding toxic shock syndrome toxin, staphylococcal enterotoxin B, and other superantigens [3]. The spread of SaPIs is mediated by the so-called helper phages through a unique mechanism in which SaPIs residing in the staphylococcal genome replicate autonomously upon helper phage invasion or prophage activation. Thereafter a specific derepressor protein of the phage relieve the repression of the genes responsible for SaPI excision, replication, and packaging [4]. 

It has been shown that in case of SaPIbov1 pathogenicity island homotrimeric dUTPase enzymes of specific phages are responsible for the SaPI induction through direct interaction with Stl; the master repressor protein of the SaPI lifecycle [5,6]. Homotrimeric phage dUTPases share a conserved core and also frequently contain an approximately 30–40-residue-long, diverse phage-specific insertion. It has been hypothesized that this phage-specific insert plays an important role in the SaPI induction since the pH15 phage dUTPase, which lacks this insertion region, cannot function as a SaPI-derepressor [5,7]. However, it has been shown that a mutant φ11 phage dUTPase lacking this phage-specific insert also interacts with the Stl, although it disrupts the Stl-DNA interaction less effectively than the wild-type protein and has a reduced capacity to induce SaPIbov1 [6,7,8]. Moreover, it was also reported that mycobacterial dUTPase lacking the phage specific insert can also bind to Stl in vitro and in vivo [9]. On the other hand, φSaov3 and φB2 phage dUTPases that contain the same sequence of the insert as the SaPI inducing dUTPases of phage φ11 and 80α, respectively, are not capable of derepression [10]. In addition, it has also been established that neither φ11 nor 80α phage dUTPases can bind to the substrate dUTP and Stl at the same time, suggesting the involvement of the dUTPase active site in the dUTPase:Stl complex formation [6,7]. The active site of trimeric dUTPases is built up as the following: A substrate binding pocket is formed by conserved motifs 1,2,4 from one of the protomers and motif 3 of a second protomer, motif 5 from the third protomer closes the active site upon substrate binding [11,12]. As dUTP hinders dUTPase:Stl complex formation, it was suggested that Stl binds to dUTPase in an open, substrate-free conformation, while it is unable to bind to dUTPase when the binding pocket is in closed conformation [6]. It has also been shown that motif 5 has negligible contribution to the protein–protein interaction in case of φ11 phage dUTPase while it has somewhat more pronounced role in case of 80α phage dUTPase [6,7]. Taking these data together it was appealing to hypothesize that the substrate binding pocket is directly involved in Stl-dUTPase interaction. Although dUTPase activity per se is not essential for SaPI mobilization there is evidence that certain mutations of specific residues in motif 4 and motif 3 influence the SaPI induction capability of 80α phage dUTPase, which argues that the dUTPase active site has key role in the complex formation with Stl [7,13]. 

**Figure 1:**
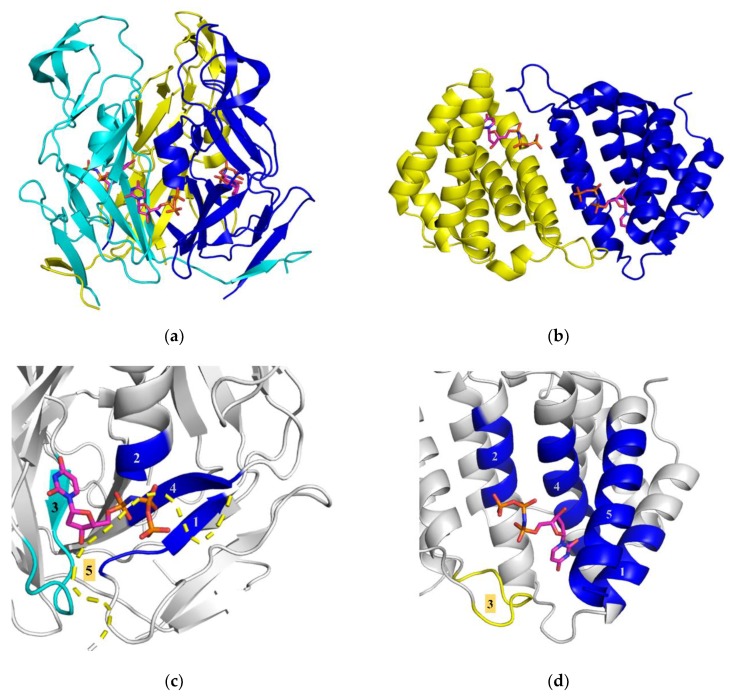
Comparison of homotrimeric and homodimeric phage dUTPases. (**a**) Structure of the β-pleated trimeric φ11 phage dUTPase (PDB ID 4GV8) protein is represented as cartoon, substrate analogue dUPNPP shown as sticks with atomic coloring as carbon: magenta, oxygen: red, nitrogen: blue, phosphorus: orange. (**b**) An all-α-helical dimeric dUTPase protein of φDI Staphylococcal phage (PDB ID 5MYD, [17]) is represented as cartoon, dUPNPP shown as sticks with atomic coloring as carbon: magenta, oxygen: red, nitrogen: blue, phosphorus: orange. (**c**) Close-up representing the architecture of the trimeric φ11 phage dUTPase active site (PDB ID 4GV8 [18], motif 5 is not localized in the φ11 phage dUTPase electron density map, so that is modeled based on the 80α phage dUTPase structure [PDB ID: 3ZEZ]). Conserved motifs are colored by subunits, substrate analogue dUPNPP shown as sticks with atomic coloring according to (a). The substrate binding pocket is constituted at the interface of two subunits by conserved motifs 1, 2, and 4 of one subunit and motif 3 of the other subunit. Upon dUTP hydrolysis the pocket is closed by motif 5 of the third subunit. (**d**) Close-up representing the architecture of the all-α-helical dimeric dUTPase protein of φDI Staphylococcal phage (PDB ID 5MYD, [17]). Conserved motifs are colored by subunits, substrate analogue dUPNPP shown as sticks with atomic coloring according to (a). The substrate binding pocket is located at the dimer interface.

In parallel to these studies it has also been revealed that not only the homotrimeric phage dUTPases but a homodimeric dUTPase from φNM1 phage is also capable to interact with the Stl of SaPIbov1 [14,15]. Hill et al. provided clear evidence also for the direct interaction of the φNM1 phage dUTPase and Stl [14]. This finding is surprising since homodimeric and homotrimeric dUTPases share no structural similarity: dimeric dUTPases are all-α helical proteins while trimeric dUTPases have a β-pleated 3D fold (Figure 1) [12,16,17]. The two active sites of dimeric dUTPases are built up symmetrically on the interface of the dimer by 5 motifs as one protomer provides motif 1,2,4,5 and the other donates motif 3. Although it has turned out that in case of φNM1 phage dUTPase the enzymatic activity is not essential for SaPI mobilization [15], the two structurally highly different dUTPase families have only the dUTP binding ability in common, so it is suggestive to speculate on the role of this region in Stl binding. 

No detailed study is yet available which investigates the peptide segments involved in the formation of the binding surface of Stl with phage dUTPases of different folds. Furthermore, the large structural differences between homotrimeric and homodimeric dUTPases poses the question whether Stl promiscuity is orchestrated by different binding peptide segments of the Stl protein or if dUTPases present the same binding surface to Stl (potentially comprising the active site where the substrate dUTP is accommodated) despite their different folds. This question was also investigated by Bowring et al. in their study published in 2017 [19] using two truncated Stl constructs: Stl-1-175 and Stl-87-267. This latter construct with two additional residues (Stl-85-276) was first characterized and termed as Stl-C-terminal domain in our previous work published in 2015 [20]. Bowring et al. concluded that these two constructs interact differently with the trimeric φ11 phage dUTPase and the dimeric φO11 phage dUTPase. We have previously shown that the Stl C-terminal domain binds to the trimeric φ11 phage dUTPase with high affinity compared to the full-length Stl, and also strongly inhibit the enzymatic activity of the enzyme [20]. The results reported by Bowring et al. [19] were in disagreement with our previous data [20]; however, the reason for this discrepancy was not addressed in [19]. 

Herein we set out to explore the binding mechanism of the interaction between homodimeric and homotrimeric dUTPases and Stl. We also aim to clarify the controversy between our previous data [20] and the study of Bowring et al. [19]. Based on enzyme inhibition assays, we here show that homodimeric φNM1 dUTPase has similar affinity to Stl as the homotrimeric φ11 dUTPase. We also show that the binding of Stl to the homodimeric φNM1 dUTPase results in dissociation of the homodimer and the formation of heterodimeric Stl:dUTPase assemblies. These events may be important for dUTPase inhibition given that the active sites of this protein are located in the dUTPase dimer interface. This is markedly different from the trimeric dUTPases which interact with Stl without the change of oligomeric state. In order to provide exclusive insight to the structural details of complex formation, we performed hydrogen deuterium exchange mass spectrometry measurements. This pioneering technique can reveal information such as the change in H/D exchange rate upon complex formation [21,22,23]. If a decrease is observed in a specific area, that region is suggested to be directly involved in the protein–protein interaction [24,25]. Based on our hydrogen deuterium exchange mass spectrometry (HDX-MS) results, we identify regions of both Stl and homotrimeric and homodimeric dUTPase proteins which are involved for complex formation.

## 2. Materials and Methods

### 2.1. Cloning, Expression, and Purification of Proteins 

The φNM1 phage dUTPase (DUTφNM1, Uniprot ID: A0EWK2, residues 2-178) was amplified from the pET21A vector provided by the courtesy of Dokland laboratory [14] using 5′-TATTGGATCCATGGCTAGCACTAACACATTAACA-3′ forward and 5′-GGTCCTCGAGTTACACGTATCCTTTTCCTGCG-3′ reverse primers and cloned to a pGEX-4T-1 vector in frame with the thrombin cleavable amino-terminal GST tag by using BAMHI and XhoI restriction sites. The resulting construct was validated by sequencing (Eurofins MWG Operon). DUTφ11 was expressed from a pET-15b plasmid created by cloning of the codon-optimized cDNA of DUTφ11 that was cloned into the vector from Novagen with *NdeI* and *XhoI* restriction sites using the services of Eurofins MWG Operon. A truncated mutant of the φ11 dUTPase lacking the phage specific insert DUTφ11^Δinsert^ and Stl were expressed from constructs designed earlier [18,26]. Sequences of the proteins are shown in Supplementary Table S1.

Proteins were expressed in *E. coli* strain BL21 Rosetta (DE3) propagated on Luria–Bertrani broth till OD_600_ = 0.6 and then induced with 5 mM isopropyl-β-D-1-thiogalactopyranoside (IPTG) for 4 h at 30 °C in case of Stl and DUTφNM1 and 37 °C for DUTφ11 and DUTφ11^Δinsert^. The cells were then harvested by centrifugation (30 min 16000*g*) and stored at −80 °C.

Purification of GST-tagged Stl and DUTφNM1 proteins were carried out as described earlier for the case of Stl [20]. Briefly, cell pellets were resuspended by Potter–Elvehjem homogenizer in 30 mL buffer A (50 mL HEPES (pH = 7.5), 200 mM NaCl) supplemented with 2 mM dithiothreitol (DTT), ca. 2 µg/mL RNase and DNase, and an EDTA-free complete ULTRA protease inhibitor tablet (Roche). The cell suspension was sonicated (4 × 60 s), and centrifuged (16000*g*, 30 min). The supernatant was loaded on a pre-equilibrated benchtop glutathione-agarose affinity-chromatography column (GE Healthcare) and then the column was washed with ten volumes of buffer A. The GST tag was removed by overnight on-column cleavage of the fusion-protein by of 80 unit thrombin (GE Healthcare) in 4 mL buffer A at 20 °C. Pure proteins (>95% as verified by SDS gel electrophoresis) were obtained in the flow-through. 

Purification of DUTφ11 was carried out by NiNTA affinity chromatography and a subsequent gel filtration as the following: protein was solubilized in 50 mL lysis buffer (50 mM TRIS·HCl, pH = 8.0, 300 mM NaCl, 0.5 mM EDTA, 0.1% Triton X-100, 10 mM 2-mercaptoethanol, 5 mM benzamidine, 1 mM PMSF; ca. 2 µg/mL RNase and DNase and an EDTA-free complete ULTRA protease inhibitor tablet (Roche)). Following 4 × 60 s sonication, the supernatant from centrifugation (16,000*g*, 30 min) was applied onto a Ni-NTA column (Novagen) pre-equilibrated with lysis buffer containing 15 mM imidazole. After removing the contaminants by washing the column with ten bed volumes of low salt and high salt buffers (50 mM HEPES pH = 7.5, supplemented with 30 mM KCl or 300 mM KCl, respectively), DUTφ11 was eluted with 500 mM imidazole dissolved in low salt buffer. After elution DUTφ11 was dialyzed against buffer B (50 mM HEPES, pH = 7.5, 300 mM NaCl, 5 mM MgCl_2_) and then gel-filtrated in buffer B on a GE Healthcare S200 Increase 10/300 (24 mL) column.Purity of the obtained protein preparation was above 95% based on SDS gel electrophoresis results.

Purification of DUTφ11^Δinsert^ was carried out as described earlier [6]. Shortly, protein was solubilized in the same way as DUTφ11 in low salt buffer (20 mM HEPES (pH = 7.5), 100 mM NaCl, 5 mM MgCl_2_, 10 mM 2-mercaptoethanol) supplemented with 2 µg/mL RNase and DNase and one tablet of Complete ULTRA EDTA-free protease inhibitor. Supernatants were directly loaded on a Q-Sepharose column (5 mL) equilibrated with low salt buffer and eluted by applying 25 mL of a linear gradient up to 1000 mM NaCl; dUTPase appeared at 0.3–0.5 M NaCl. The second purification step was gel-filtration performed as in the case of DUTφ11. The purified DUTφ11^Δinsert^ appeared as single bands of at least 95% purity on SDS-PAGE. 

All protein preparations were either used freshly or frozen in liquid nitrogen, and stored at −80 °C in small aliquots. Concentration of the proteins was determined based on the absorbance value measured at 280 nm by NanoDrop 2000 UV-Vis spectrophotometer using the extinction coefficients calculated based on amino acid composition (http://web.expasy.org/protparam) (Supplementary Table S1).

### 2.2. dUTPase Enzyme Activity Assay

Proton release during the transformation of dUTP into dUMP and PPi was followed using a Jasco V550 spectrophotometer at 559 nm and 293 K. Reaction mixtures contained DUTφNM1 dUTPase enzyme and Stl protein at different concentrations of 0–300 nM in 1 mM HEPES–HCl (pH = 7.5) buffer containing 5 mM MgCl_2_, 150 mM KCl, and 40 mM phenol red pH indicator. The reaction was initialized with 30 mM dUTP after pre-incubation of proteins for 5 min. The initial velocity was determined from the slope of the first 10% of the progress curve. Quadratic binding equation was fitted to the data.

### 2.3. Native Gel Electrophoresis

**Figure 2:**
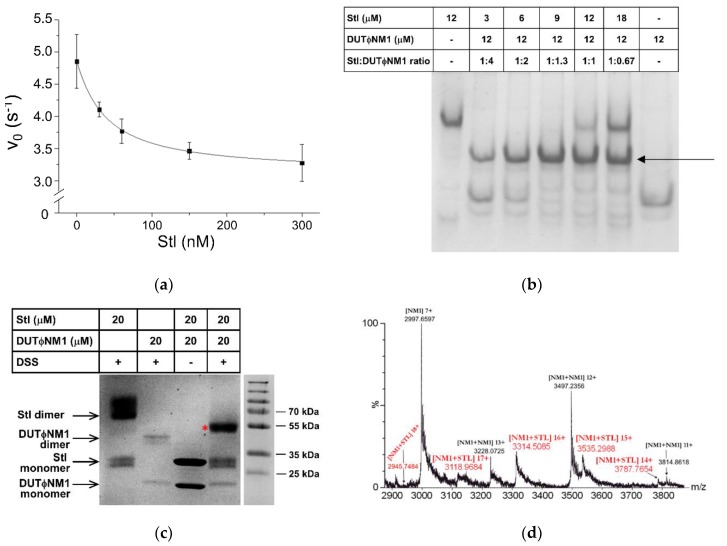
Biophysical characterization of φNM1 phage dUTPase-Stl interaction. (**a**) Enzyme activity of homodimeric DUTφNM1 in the presence and absence of Stl (steady-state conditions). The maximal extent of inhibition was about 40%, the binding is characterized by the apparent inhibitory constant of K_i, app_ = 34 ± 14 nM. (**b**) Native gel electrophoresis of Stl, DUTφNM1 and the mixture of the two proteins of various molar ratios, species, and concentrations of monomers are indicated in the figure. Note the emerging band (indicated by arrow) in the samples containing the mixture of the two proteins that suggests the formation of an Stl-DUTφNM1 heterodimer, situated between the bands of the individual dimeric proteins. (**c**) SDS-PAGE analysis of Stl, DUTφNM1, and their mixture after chemical crosslinking induced by the reagent disuccinimidyl suberate (DSS). The mixture of the untreated proteins was also loaded on the gel as a control. The band corresponding to the molecular mass of the DUTφNM1-Stl heterodimer is denoted with a red star. (**d**) The native mass spectrum of the DUTφNM1-Stl mixture. Peak series with *m*/*z* values of 2946 (18+), 3119 (17+), 3315 (16+), 3535 (15+), 3788 (14+) (highlighted in red) indicate the presence of an assembly associated with the molar mass of 53020 ± 7 Da, which corresponds to a 1:1 complex of Stl (32.0 kDa) and DUTφNM1 (21.0 kDa), constituting the DUTφNM1-Stl heterodimer (designated as “NM1+Stl” on the figure). Peaks corresponding to DUTφNM1 monomer (NM1) and homodimer (NM1+NM1) are also present in the spectrum.

Native gel electrophoresis was performed in 12% polyacrylamide gel. After 1-h pre-electrophoresis with constant voltages of 100 V in Tris-HCl buffer (pH = 8.7), 15 μL of the premixed samples was loaded onto the gel and electrophoresed for 1.5 h on 150 V. In order to avoid protein denaturation the apparatus was cooled on ice during procedure. Gels were stained by Page Blue protein staining solution (Thermo Fisher). Species and concentrations of monomers are indicated on Figure 2b.

### 2.4. Chemical Crosslinking

Stl and DUTφNM1 samples of 20 μM concentration and the dUTPase-Stl mixtures of 1:1 molar ratio (40 μM total protein concentration) were prepared and incubated for 5 min at 20 °C, then 20 mM disuccinimidyl suberate (DSS) was added to the samples, followed by a further incubation at 20 °C for 1 h. Quenching of the crosslinking reaction was performed by the addition of 5 μL 100 mM (pH = 7.5) Tris buffer to 40 μL of samples and were analyzed by SDS-PAGE on a 12% gel using Page Ruler prestained protein ladder as a molecular weight marker. Gels were stained by Page Blue protein staining solution (Thermo Fisher).

### 2.5. Native Mass Spectrometry

For the mass spectrometry measurements of DUTφNM1 and the DUTφNM1:Stl complex, a commercial Waters QTOF Premier instrument equipped with an electrospray ionization source was used in positive ion mode. The mass spectra were recorded under native conditions, and the mixtures contained the proteins at concentration of 40 μM in 5 mM NH_4_HCO_3_ buffer solution (pH = 8.0). These conditions allow transfer of the native protein complexes to the gas phase. The capillary voltage was 2600 V, the sampling cone voltage was 128 V, and the temperature of the source was kept at 363 K. Mass spectra were obtained in the mass range of 1500–6000 *m*/*z*.

### 2.6. HDX-MS

HDX-MS acquisitions were performed on a Synapt G2Si HDMS coupled to an Acquity UPLC M-Class system with HDX and automation (Waters Corporation, Manchester, UK). The deuterium uptake of the DUTφ11, DUTφNM1, and Stl proteins was determined using a continuous workflow with labelling taking place at 20 °C. Each protein was solubilized in Buffer S (20 mM HEPES, 300 mM NaCl, 5 mM MgCl_2_, pH = 7.5) to a working concentration of 10–20 µM. Deuterium labelling was initiated by diluting 5 μL of each protein sample into 95 μL of Buffer L (20 mM HEPES, 300 mM NaCl, 5 mM MgCl_2_ in D_2_O, pD = 7.1). After various incubation times, samples were quenched in Buffer Q (2.4% formic acid) at 1 °C to retard further deuteration or back-exchange and were then digested on-line with a Waters Enzymate BEH pepsin column at 20 °C. Trapping of the peptides occurred on a Waters BEH C18 VanGuard pre-column for 3 min at a flow rate of 200 μL/min in 0.1% formic acid (pH = 2.5) before being applied to a Waters BEH C-18 analytical column. Elution of the peptides was achieved using a linear gradient of Buffer E (0.1% formic acid in acetonitrile, pH = 2.5) at a flow rate of 40 μL/min. To minimize back-exchange all trapping and chromatography stages of the experiment are performed at 0.5 °C. Determination of the bound HDX profile of each protein was carried out by pre-mixing the proteins at approximately equimolar concentrations. MS data were acquired using an MSE workflow in HD mode with extended range enabled to reduce the detector saturation and maintain peak shapes. Undeuterated reference acquisitions were obtained in sextuplicate for each protein along with labelling acquisitions of 1, 10, and 100 min, which were obtained in triplicate. The MS was calibrated using NaI and MS data were obtained with lock mass correction using Leu-enkephalin.

Peptides were assigned with the ProteinLynx Global Server (PLGS) (Waters Corporation, Manchester, UK) software package, with the deuterium uptake of each assigned peptide being determined with DynamX v3.0 (Waters Corporation, Manchester, UK). Evaluation of the data fitting as well as determining the error of each dataset were performed as previously described [27]. The total Δmass of each peptide was then plotted against the residue position, allowing the generation of “Woods plots” which describe the Δmass of each peptide in the bound state [28]. The average Δmass across all peptides at each residue was then calculated. Residues with values exceeding the 99% confidence bands are noted and defined as part of the interaction surface of Stl and phage dUTPases. In all cases sequence coverage was above 90% and redundancy was above 3.

### 2.7. Homology Models

In case of Stl the formerly generated and validated Phyre2 model was used [20,26], 3D homology model of DUTφNM1 was created also with Phyre2 based on the crystal structure of φDI and φO11 phage dUTPases (PDB ID: 5MYD, 5MIL) [17,19,29].

## 3. Results and Discussion

### 3.1. Stl Inhibits the Enzymatic Activity of Homodimeric and Homotrimeric dUTPases with Comparable Inhibition Constant

It has been shown that the homodimeric DUTφNM1 induces the replication of SaPIbov1 through complex formation with Stl, and this complex formation also results in the decrease of enzymatic activity of the DUTφNM1 enzyme [14]. In the present study we quantitatively analyzed the inhibition of DUTφNM1 by Stl (Figure 2a). Based on steady-state enzymatic activity measurements of DUTφNM1 performed in the presence of Stl of different concentrations, we found that the maximal inhibition was about 40%, thus half of the original enzymatic activity was retained even at relatively high concentration of Stl. This markedly differs from the complete loss of dUTPase enzymatic activity observed upon homotrimeric DUTφ11-Stl complex formation within the same steady-state assay conditions [6]. This result on its own does not necessarily implicate weaker binding per se, as for example in case of competitive inhibition observed for the DUTφ11-Stl system [6], the extent of inhibitory effect on enzymatic activity is determined by the dissociation and association kinetics of both the inhibitor and the ligand. Indeed, the apparent inhibitory constant found in case of the homotrimeric DUTφ11 (K_i, app_ = 27 ± 5 nM, c.f. [6]), is comparable to the data we obtained here for the homodimeric DUTφNM1, K_i, app_ = 34 ± 14 nM (Figure 2a). The exact mechanism of inhibition can only be revealed by detailed transient kinetic and thermodynamic characterization of the dimerization and substrate binding of the DUTφNM1 in the presence and absence of Stl, which was beyond the scope of this study.

### 3.2. Mechanism of Interaction with Stl is Markedly Different between Homodimeric and Homotrimeric Phage dUTPases

The stoichiometry of the DUTφNM1-Stl complex was then investigated using various biochemical and biophysical assays to critically evaluate and expand the suggestion for the existence of a heterodimer based on the chemical crosslinking by Hill and Dokland [14]. The proteins were first characterized by native gel electrophoresis on their own or premixed (Figure 2b). Mixtures of the Stl and DUTφNM1 proteins represented different ratios of the proteins in the samples (see numbers of ratios and concentrations of monomers above the specific lanes on Figure 2b). Lanes containing the individual proteins (either DUTφNM1 or Stl on their own) show bands corresponding to the homodimeric assemblies as previously described [6,14]. Upon mixing the two proteins, a new third band clearly emerged, that was not present in the samples of the individual proteins (highlighted with an arrow on the figure). The presence of this new band argues for the formation of a DUTφNM1-Stl complex. 

To confirm the complex formation and investigate its stoichiometry, the assemblies were then investigated by chemical crosslinking (Figure 2c). Previous cross linking experiments have reported a 1:1 DUTφNM1-Stl heterodimer [14], although the short spacers (ca. 5 Å) used in these experiments may have resulted in overrepresentation of these assemblies. We performed the crosslinking of the proteins by using disuccinimidyl suberate (DSS) which possesses a ca. 11 Å linker distance in order to identify any higher order complex of DUTφNM1 and Stl. SDS-PAGE analysis of the individual proteins after crosslinking resulted in the presence of two bands for each protein with molecular weights corresponding to those expected for the monomeric and dimeric proteins (Figure 2c). In the premixed samples containing a mixture of DUTφNM1 and Stl in 1:1 molar ratio a unique band is present with a molecular weight (ca. 55 kDa) consistent with that expected for a DUTφNM1:Stl heterodimer in accordance with the native gel electrophoresis experiments. As we have not found any other assembly of higher molecular weight we also concluded that the DUTφNM1-Stl complex is likely to consist of one monomer of Stl and one monomer of DUTφNM1.

The 1:1 composition of the DUTφNM1–Stl complex was also confirmed by native mass-spectrometry measurements (Figure 2d, Figure S1). In the spectrum, the monomer form of the DUTφNM1 protein was the most abundant showing a Gaussian-like distribution of the *m*/*z* values 1907.934 (+11), 2098.611 (+10), 2331.718 (+9), 2623.025 (+8), 2997.660 (+7), 3497.052 (+6), 4196.261 (+5), 5245.075 (+4) (Figure S1a). The mass calculated from these MS peaks is 20976 Da, which corresponds to the molar mass of 20976 Da calculated based on amino acid composition of the protein (http://web.expasy.org/protparam) (Supplementary Table S1). Although less abundant, still the peaks corresponding to a dimer of DUTφNM1 were also observable in the spectrum as series of peaks with *m*/*z* 3228.073 (+13), 3497.235 (+12), 3814.862 (+11) associated with the molar mass of 41950 Da, well agreeing with the expected 41952 Da for a homodimer of DUTφNM1 (Figure S1a). New peaks of *m*/*z* 2945.7484 (+18), 3118.9684 (+17), 3314.5295 (+16), 3533.9619 (+15), 3787.103 (+14), 4078.3411 (+13), 4418.1189 (+12), 4819.6745 (+11) were also emerging in the spectra of the mixture of the DUTφNM1 and Stl. These peaks are associated with the molar mass of 53020 Da. This clearly shows that the complex consists of one monomer Stl (32016 Da) and one monomer DUTφNM1 (20976 Da), i.e., 1:1 stoichiometry is observed.

In contrast to this, the stoichiometry of the complex resulting from the interaction of the homotrimeric DUTφ11 with Stl was found to be 3:2 or 3:3 dUTPase: Stl [6,8], suggesting that DUTφ11 remains in homotrimeric oligomeric state upon complex formation. Small-angle X-ray scattering studies suggested that in the trimeric human dUTPase-Stl complex, Stl is present in monomers [26]. Here we observe that in the interaction of the DUTφNM1 protein with Stl, both proteins dissociate into monomers and form a heterodimer. These results can provide a possible explanation on the mechanism of enzymatic inhibition of the DUTφNM1 protein, since in case of DUTφNM1, the active site is located at the dimer interface of the protein (cf. Figure 1), which is likely affected by the Stl binding. It has been suggested that Stl as other similar repressors binds to its cognate DNA site as a dimer. Complex formation with either DUTφNM1 of DUTφ11 involves monomers of Stl (cf. [6,7,26] and present work), which provides a potential model for the perturbation of the Stl-DNA complex through dissociation of the repressor dimers to interact with the derepressor in both types of complexes. 

### 3.3. dUTPase Active Sites are Directly Involved in the Complex Formation with Stl

The protein surfaces responsible for the interaction between DUTφNM1:Stl and DUTφ11:Stl complexes were investigated using hydrogen deuterium exchange mass spectrometry (HDX-MS), similarly as in a previous study [26]. This method reports on a protonated protein’s time-dependent uptake of deuterium when dissolved in a fully deuterated solvent, in which changes can be localized to peptide units across the protein backbone. In binding assays, HDX-MS outputs are typically reported by changes in the rate of isotope uptake (Δmass) between unbound and bound protein complexes, yielding characteristic difference plots that provide unique insight into protein–protein interfaces [23]. In the present study, we determined HDX-MS data for all proteins either in isolation or in premixed samples and then difference plots were prepared by subtraction of the uptake data obtained for the bound protein complexes from those obtained for the proteins in their unbound conformations (cf. Methods).

**Figure 3:**
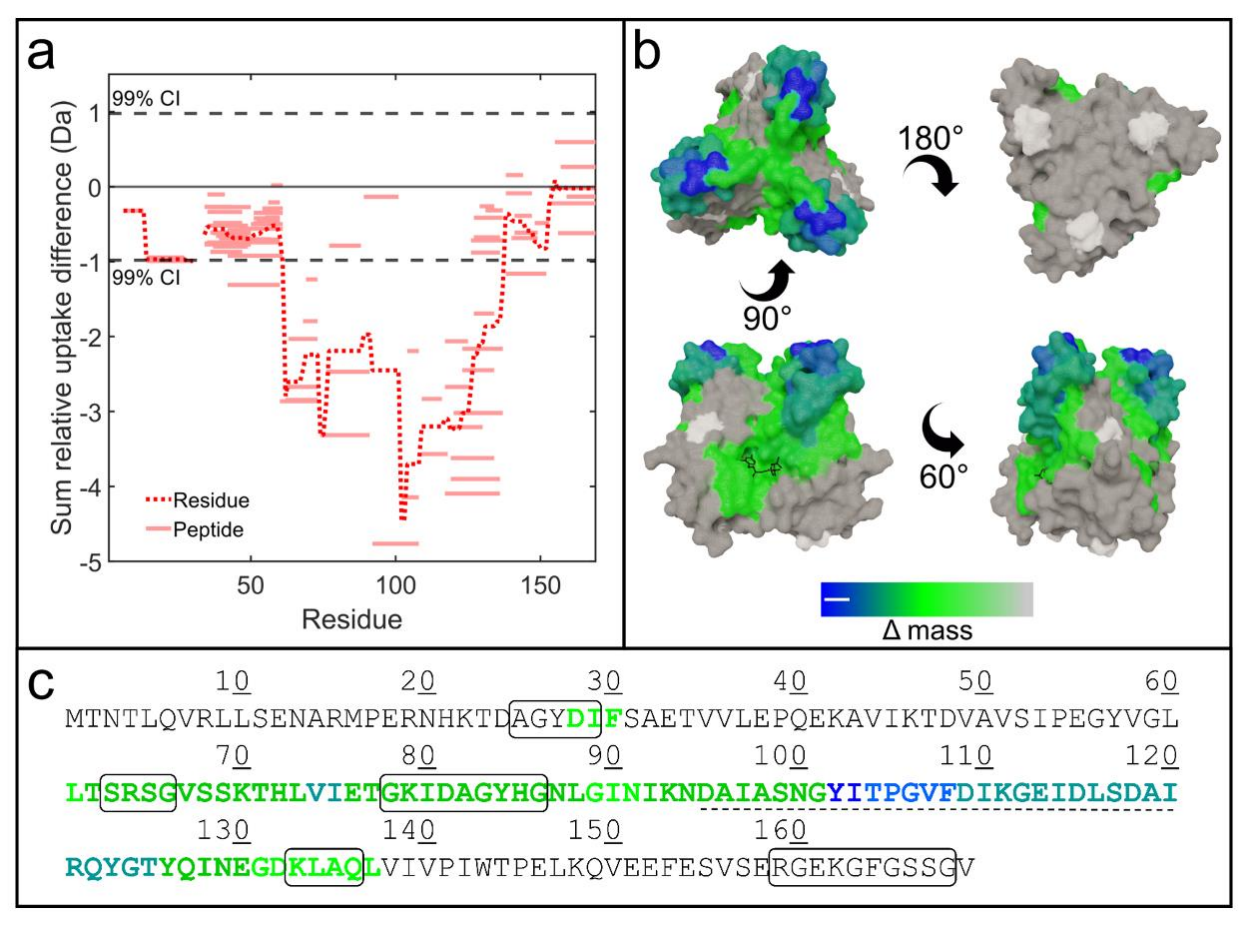
Hydrogen deuterium exchange mass spectrometry (HDX-MS) results for DUTφ11 upon mixing with Stl. (**a**) HDX difference plot showing the Δmass of each peptide (solid lines) as well as the average amount per residue (dotted line). The dashed lines represent the 99% confidence bands evaluated over the whole dataset. (**b**) X-ray crystal structure (PDB ID 4GV8 [23]) of DUTφ11 colored according to HDX-MS difference data following the color gradient shown at the bottom of the panel; substrate analogue dUPNPP is also shown as black sticks in order to ease visualization of the active sites (views: top, bottom, sides). Regions which could not be probed by HDX-MS are shown in white. (**c**) Sequence of the DUTφ11 is shown, where the conserved active site building motifs are boxed and the phage specific insert is underlined with a dashed line. Letter coloring is according to HDX difference data.

**Figure 4:**
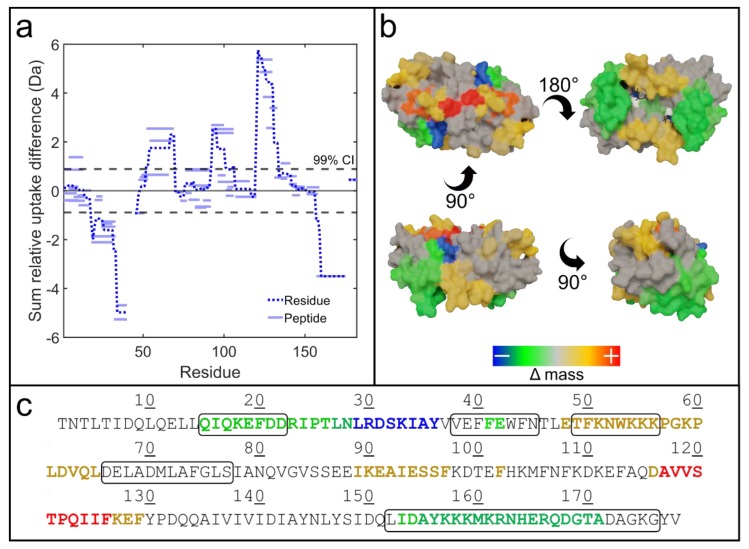
HDX-MS results for DUTφNM1 upon mixing with Stl. (**a**) HDX difference plot showing the Δmass of each peptide (solid lines) as well as the average amount per residue (dotted line). The dashed lines represent the 99% confidence bands evaluated over the whole dataset. (**b**) Homology model of DUTφNM1 colored according to HDX-MS difference data following the color gradient shown at the bottom of the panel; substrate analogue dUPNPP is also shown as black sticks in order to ease the visualization of the active sites (views: top, bottom, sides). Regions which could not be probed by HDX-MS are shown in white. (**c**) Sequence of the DUTφNM1 protein. Conserved active site building motifs are boxed, letter coloring is according to HDX difference data.

The HDX-MS difference plots of both dimeric and trimeric dUTPases exhibited large changes in isotope uptake in the presence of Stl consistent with the binding of the inhibitor to these proteins (Figure 3 and Figure 4). In the case of DUTφ11, the most conspicuous Δmass data were observed for peptides that spanned most of the active site segments as well as the phage specific insert of the protein (Figure 3, peptide numbering is shown on Figure S2). This indicates the direct involvement of DUTφ11 active site in the complex formation with Stl (cf. also Supplementary Figure S3) and is consistent with previous work showing that the dUTP substrate and the Stl compete for the same binding site [6]. Similar conclusions were drawn for the trimeric human dUTPase by HDX-MS [26] and for the trimeric dUTPase from *E. coli* by mutational analysis [30]. So that it seems that Stl may have a uniform binding mode to the trimeric dUTPases. As it has been shown that the phage specific insert is not essential for the binding of DUTφ11 to Stl [8], possibly the interaction of the residues of the insert with Stl is the consequence of binding of the inhibitor protein to the active site of DUTφ11. This hypothesis has been reinforced by the HDX-MS data obtained for a truncated mutant, which lacks the phage specific insert, DUTφ11^Δinsert^ (Figure S2 and S4–S6). In the experiments with this mutant protein and Stl, the large Δmass data for the dUTPase active site segments were clearly preserved (cf. Figure S5b).

It is important to note that no significant Δmass was observed for the DUTφ11 conserved motif 5. This finding is also in agreement with the previous results indicating that the truncated mutant of the DUTφ11 protein lacking motif 5 was capable of binding to Stl with similar affinity as that of the full length, wild-type protein [6,7].

The HDX-MS results for the DUTφNM1–Stl complexes revealed significantly more complex binding interaction as compared with the outputs of DUTφ11-Stl (Figure 4, peptide numbering is shown on Figure S6). Peptides spanning residues 15–36 and 155–171 of DUTφNM1 show significant decreases in Δmass consistent with the binding of Stl and occlusion of these sites from isotope exchange. These regions contain active site residues of potential key-importance in enzymatic activity including Q17, D21 (residues of motif 1 responsible for uracil binding), K159 and R166 (residues of motif 5 responsible for phosphate binding) as determined by sequence alignment against a dimeric dUTPase with detailed study on the active site [31], and dimeric phage dUTPase structures [17,19] (Figure 4b). This suggests that part of the active site is directly involved in the protein–protein complex formation, as these residues of the protein become less accessible to the solvent upon complex formation (cf. also Supplementary Figure S2). However, we also note here that specific mutation of K159 to alanine did not abolish DUTφNM1:Stl complex formation either in vitro or in vivo, thus this residue is not an essential factor in the protein–protein interaction [15]. 

In the presence of Stl, the DUTφNM1 protein also shows extended regions of positive Δmass including peptides covering residues 48–65, 89–97, and 116–129. The results potentially indicate that these protein regions may become more solvent accessible in the presence of Stl presumably as a consequence of dimer dissociation as observed in the crosslinking assay, or undergo other conformational changes. To better understand the significance of these results, the HDX-MS outputs were mapped onto a 3D homology model of DUTφNM1 dimer (Figure 4c). According to the analysis of residue–residue interactions across the dimer interface of the in silico 3D model performed with DIMPLOT [32], residues 45–58 and 111–124 are located on the dimer interface. Thus, the increase in H/D exchange rate detected in these regions might correspond to the increased solvent accessibility of these segments because of the dissociation of DUTφNM1 homodimer upon complex formation with Stl. All in all, the native gel electrophoresis, chemical crosslinking, enzyme activity, native MS, and HDX-MS experiments all support the formation of a heterodimer complex constituting DUTφNM1 and Stl.

It has been formerly proposed that a short segment with common sequence (GVSS) of the DUTφ11 and DUTφNM1 might have a role in complex formation [15] (cf. also sequence alignment on Supplementary Figure S9). Although this segment of DUTφ11 (residues 66–69) showed significant decrease in H/D exchange rate, the same segment of DUTφNM1 (residues 83–86) did not present significant HDX signal upon complex formation, arguing against this hypothesis. Further analysis is required to decide on the potential role of this segment in complex formation.

### 3.4. Different Regions of Stl Mediate the Promiscuity of this Protein for dUTPase Binding 

**Figure 5:**
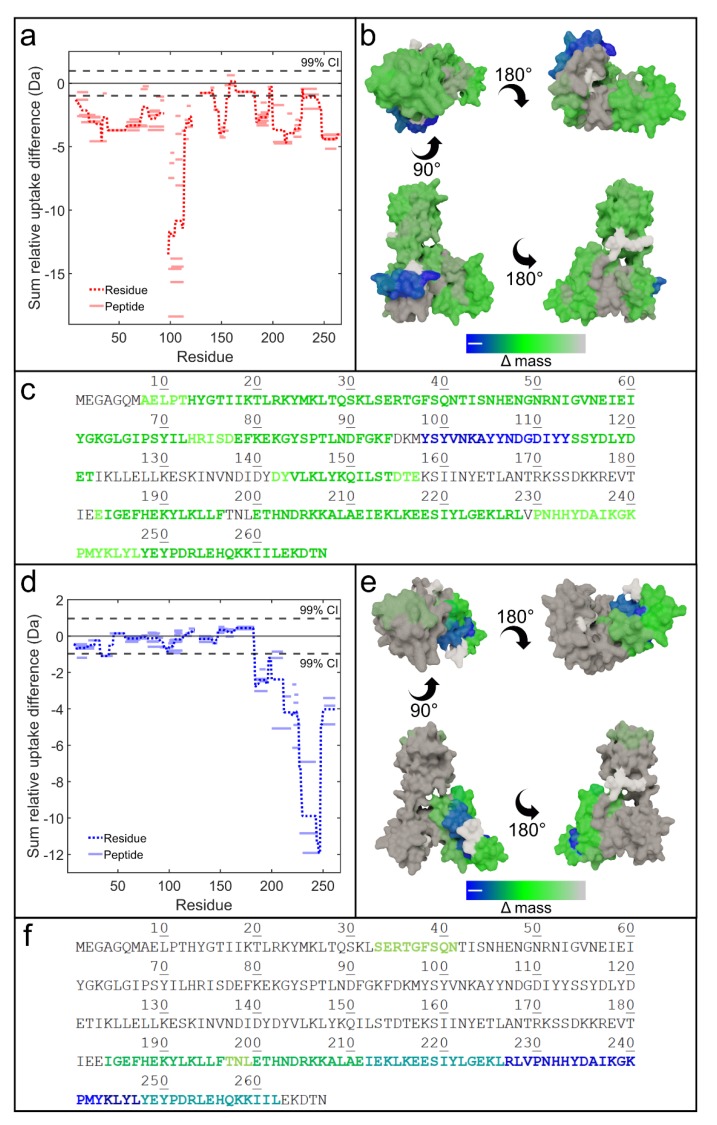
Experimental results and schematic models showing how the Stl repressor protein of SaPIbov1 interacts with drastically different families of phage dUTPases. (**a**,**d**) HDX difference plots showing the Δmass of each peptide (solid lines) as well as the average amount per residue (dotted lines) of Stl upon mixing with DUTφ11 (a) and DUTφNM1 (d). The dashed lines represent the 99% confidence bands evaluated over the whole dataset. (**b**,**e**) Homology model of Stl colored according to the HDX-MS difference data obtained upon binding to DUTφ11 (b) and DUTφNM1 (e), following the color gradient shown at the bottom of the panel (views: top, bottom, sides). Regions which could not be probed by HDX-MS are shown in white. (**c**,**f**) Sequence of Stl is shown with letter coloring according to HDX difference data when bound to DUTφ11 (c) and DUTφNM1 (f).

We next compared the different plots of Stl in the presence of the dUTPases representing the homodimeric and homotrimeric families in order to better understand the interesting capability of Stl to interact with both types of dUTPases (cf. Figure 5, peptide numbering is shown on Figures S4 and S8). These experiments were also expected to shed light on the contradiction between the data published by Nyiri et al. in 2015 [20] and Bowring et al. in 2017 [19] for interaction of Stl-C-terminal domain (Stl-85-267) with DUTφ11. The HDX-MS outputs revealed dramatically different Δmass profiles for Stl, depending on which dUTPase is added. In the presence of DUTφ11, Stl exhibits significant negative mass shifts across the protein backbone. This suggests that the binding of Stl to DUTφ11 induces a global conformational tightening of the protein and also implies that Stl protein has a larger conformational space in the absence of dUTPase. In addition to these global changes in protein conformation, Stl also displays a dramatically pronounced negative mass shift localized to the protein region of residues ca. 98Y – 113Y (Stl-98-113). This suggests that this region plays a major role in the interaction of Stl with DUTφ11. Since this tyrosine-rich region is part of the Stl-85-267 (termed as Stl-C-terminal domain (or Stl-87-267, termed as Stl^ΔHTH^) truncated constructs, these HDX-MS data are consistent with our previous results which showed that this truncated Stl construct lacking the N-terminal 84 residues is fully capable of binding to and inhibiting the enzymatic activity of the DUTφ11 enzyme [20]. It is also of interest to point out that the very same peptide segment was also shown to be similarly involved in the interaction of Stl with human dUTPase, another trimeric dUTPase [26], strengthening the role of this segment in interactions with representatives from this family of dUTPases.

As we also showed earlier, the N-terminal 84 residues contains the DNA-binding helix-turn-helix motif of the repressor protein [20]. So the results presented in this study reinforce the suggestion that DNA-binding and protein–binding functions of Stl can be associated with different segments of the protein.

In case of the DUTφNM1-Stl complex, peptides covering the region of the N-terminal 200 residues of Stl were identified with no significant HDX change, however peptides from the 60 residue-long segment situated at the very C-terminal part of the Stl sequence showed pronounced negative signal. Within the region, the segment of Stl-227-247 shows the largest shifts. On the one hand, these results are consistent with the former finding that truncation of the N-terminal 84 residues did not perturb the complex formation between Stl and DUTφNM1 [15] or Stl and another dimeric phage dUTPase from phage φO11 [19]. On the other hand, these results also clearly delineate the different segments of the Stl-C-terminal domain that are used by the repressor protein for complex formation with DUTφ11 and human dUTPase [26] (homotrimeric dUTPase family) or DUTφNM1 (homodimeric dUTPase family).

## 5. Conclusions

Altogether, our kinetic, cross-linking, native mass spectrometry, and HDX-MS experiments suggest previously unreported functional plasticity of *S. aureus* pathogenicity island repressor protein Stl and revealed new details for a better understanding of the different binding mechanisms of Stl for the two different phage dUTPases. The HDX-MS experiments shown here suggested highly different interaction surface of Stl with the dimeric DUTφNM1 and trimeric DUTφ11 dUTPases. Native mass spectrometry data here and in earlier papers [6,26,33] provided direct experimental data for the distinct Stl-binding mechanisms of the two dUTPase families. The two families of dUTPases have evolved separately and constitute drastically different protein folds and active site architecture, as reviewed in [12,34]. It is of interest to consider that Staphylococcal phages encode dUTPase representatives from both families (cf. [6]and [19]) such that the presence of a dUTPase is a conserved character of these phages. 

Why do phages encode their own dUTPases? A reason for this may arise from the interesting lack of endogenous dUTPase from *S. aureus* strains [35]. Since dUTPase is important for preventive DNA repair [11], phages may increase their chances by encoding their own copy of this important enzyme, either from the trimeric or from the dimeric dUTPase family. SaPIs on the other hand have evolved to rely on phages for their life cycle. The Stl repressor of SaPIbov1 has been adapted to interact with both types of dUTPases that will function as derepressors of Stl function, allowing induction of the pathogenicity island mobile genetic elements (SaPIs). In this case, the conserved presence of dUTPases within the phages is also profitable for SaPIbov1. The fact that HDX-MS data showed that different segments of this Stl repressor target phages encoding different dUTPases underlines the suggestion that mobile genetic elements may gain benefit from conditions of low dUTP levels to ensure uracil-free DNA environment. One possible advantage is that the low dUTP level, provided by the dUTPase enzymatic action, enhances the fidelity of SaPI replication via diminution of the mutation rate [36], while reducing the potential for the selective evolution of phages to escape SaPI interference in parallel. 

It is especially interesting that even if integrated prophages contain the dUTPase gene, the expression of the protein is most likely being repressed [6]. In some of the *S. aureus* strains a specific inhibitor protein of the uracil DNA glycosylase, namely SaUGI presumably moderates uracil excision, while the survival of other strains is yet unexplained [35,37,38]. It has also been demonstrated that the prophage free *S. aureus* RN450 strain, which does not contain the dUTPase gene possesses elevated genomic uracil content compared to *dut+ ung+* bacteria [35]. It seems likely that mobile genetic elements may encode either dUTPase or SaUGI to escape the damaging uracil-DNA repair, which can impair their horizontal gene transfer [9]. In addition to this, uracil content of a mobile genetic element might also prevent its integration into the genome of the new host, as it was recently demonstrated in the case of human immunodeficiency virus [39,40]. It is also tempting to hypothesize that some SaPIs recognize phages through dUTPase-Stl interaction, in order to ensure their uracil-free replication [6]. This could have a dual advantage: i) Replicated SaPI genomes are not fragmented by the host repair mechanism, ii) mutation rate of phages is not elevated by the damaging base excision repair (BER), which hinder their ability to escape the SaPI interference.

**Scheme 1:**
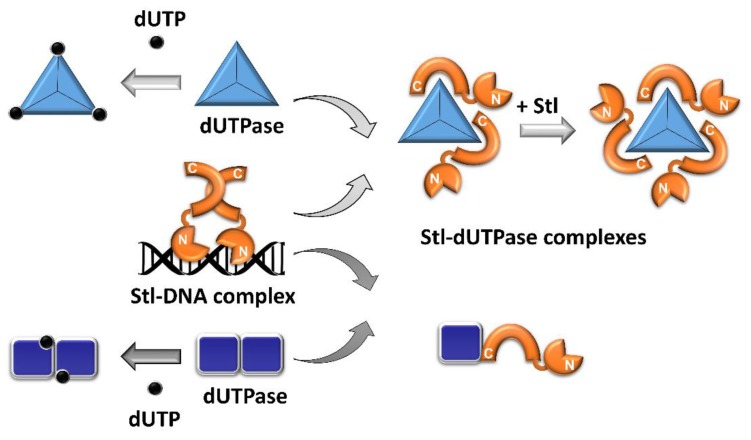
Interaction of the Stl protein with dimeric and trimeric dUTPases. Stl (orange, N-terminus is denoted with letter N, C-terminal with letter C) forms dimers in solution and may bind to DNA (black) as dimers. Perturbation of Stl dimerization by dUTPases leads to the dissociation of the Stl-DNA complex. Stl inhibits both the trimeric (light blue triangles) and dimeric dUTPases (dark blue rectangles). The inhibition is based on competition between Stl and the substrate, dUTP (black dots) in case of the trimeric dUTPases. Stl monomers and dUTPase trimers form DUT_3_Stl_2_ and DUT_3_Stl_3_ complexes. We found that the region of residues ca. 98Y – 113Y of Stl protein has a major contribution in the interaction of Stl with trimeric dUTPases (cf. also [26]). The substrate binding site of dimeric dUTPases, which is located at the dimerization surface of the enzyme, is impaired upon formation of a heterodimeric complex of the dUTPase with Stl. This explains the reduction of the enzymatic activity of dimeric dUTPases in the presence of Stl. We found that peptides from the 60 residue-long segment situated at the very C-terminal part of the Stl sequence play a key role in the heterodimer formation.

Based on our results Scheme 1 shows a schematic model, which describes the interaction of Stl with dimeric and trimeric dUTPases.

Stl protein dimerizes in solution and based on the similarity with other repressors and the symmetry of the specific binding site of the protein within the SaPI DNA, it is assumed that Stl binds to DNA as dimers (Scheme 1) [26,41]. Interaction of Stl monomers with dUTPases perturbs the dimerization of the repressor, hence it leads to the dissociation of the Stl-DNA complex. Trimeric DUTφ11 dUTPase can form DUT_3_Stl_2_ and DUT_3_Stl_3_ complexes with Stl, while in the case of dimeric enzyme DUTφNM1 the complex is a DUT-Stl heterodimer (Scheme 1) [6,9,26,33]. Based on our results Stl binds directly to the active site of trimeric dUTPases and it acts as a competitive inhibitor of these enzymes [6]. As also presented herein, Stl also reduces the enzymatic activity of dimeric dUTPases, although via a mechanism somewhat different from that observed for the trimeric enzymes. We show direct evidence from native mass spectrometry that inhibition of dimeric dUTPases by Stl during complex formation between the two proteins results from perturbation of the active site architecture, which resides at the dimer interface of the enzyme. 

## Supplementary Materials

The following are available online at https://www.mdpi.com/2218-273X/9/9/488/s1, Figure S1: Annotated mass spectra of the mixture of the DUTφNM1 (NM1) with Stl (STL) protein (a) and Stl protein (b) measured under native electrospray conditions. Figure S2: Coverage map of HDX-MS difference plots for DUTφ11 and DUTφ11^Δinsert^ upon complex formation with Stl. Figure S3: Active sites of DUTφ11 and DUTφNM1 colored according to HDX-MS difference data obtained upon complex formation of those with Stl protein. Figure S4: Coverage map of HDX-MS difference plots for Stl upon complex formation with DUTφ11 and DUTφ11^Δinsert^. Figure S5: HDX-MS difference data obtained for DUTφ11 and DUTφ11^Δinsert^ upon complex formation with Stl. Figure S6: HDX-MS difference data obtained for Stl upon complex formation with DUTφ11 and DUTφ11^Δinsert^. Figure S7: Coverage map of HDX-MS difference plots for DUTφNM1 upon complex formation with Stl. Figure S8: Coverage map of HDX-MS difference plots for Stl upon complex formation with DUTφNM1. Figure S9: Sequence alignment of φ11 phage trimeric dUTPase and φNM1 phage dimeric dUTPase generated by NPS@ server. Table S1: Sequences of protein constructs used.

## Note Added in Proof

After submission of our manuscript a relevant paper has been published (R. Ciges-Tomas et al. *Nat. Commun.* 2019, *10*, 3676.), where using different independent methods similar results were reached reinforcing the conclusions of both studies.
