# Supplementary material for: HDX and Native Mass Spectrometry Reveals the Different Structural Basis for Interaction of the Staphylococcal Pathogenicity Island Repressor Stl with Dimeric and Trimeric Phage dUTPases
Source: Biomolecules. 2019 Sep 14;9(9):488. doi: 10.3390/biom9090488 (PMC6770826; doi:10.3390/biom9090488)
Supplement: Supplementary file 1 [file biomolecules-09-00488-s001.pdf]

biomolecules

# Supplementary Materials

# HDX and native mass spectrometry reveals the different structural basis for interaction of the staphylococcal pathogenicity island repressor Stl with dimeric and trimeric phage dUTPases

**Kinga Nyíri [1,2,#,*], Matthew J. Harris [3,#], Judit Matejka [1,2], Olivér Ozohanics [4,5], Károly Vékey [4], Antoni J. Borysik [3,*], Beáta G. Vértessy [1,2,*]**

[1] Department of Applied Biotechnology and Food Sciences, Budapest University of Technology and Economics, Budapest, 1111, Hungary
[2] Institute of Enzymology, Research Centre for Natural Sciences, Hungarian Academy of Sciences, Budapest, 1117, Hungary
[3] Department of Chemistry, King's College London, Britannia House, London SE1 1DB, United Kingdom
[4] Institute of Organic Chemistry, Research Centre for Natural Sciences, Hungarian Academy of Sciences, Budapest, 1117, Hungary
[5] Department of Medical Biochemistry, Semmelweis University, Budapest, 1094, Hungary

[#] These authors contributed equally to this work.
[*] Correspondence: knyiri@mail.bme.hu; antoni.borysik@kcl.ac.uk; vertessy@mail.bme.hu

## Supplementary Figures

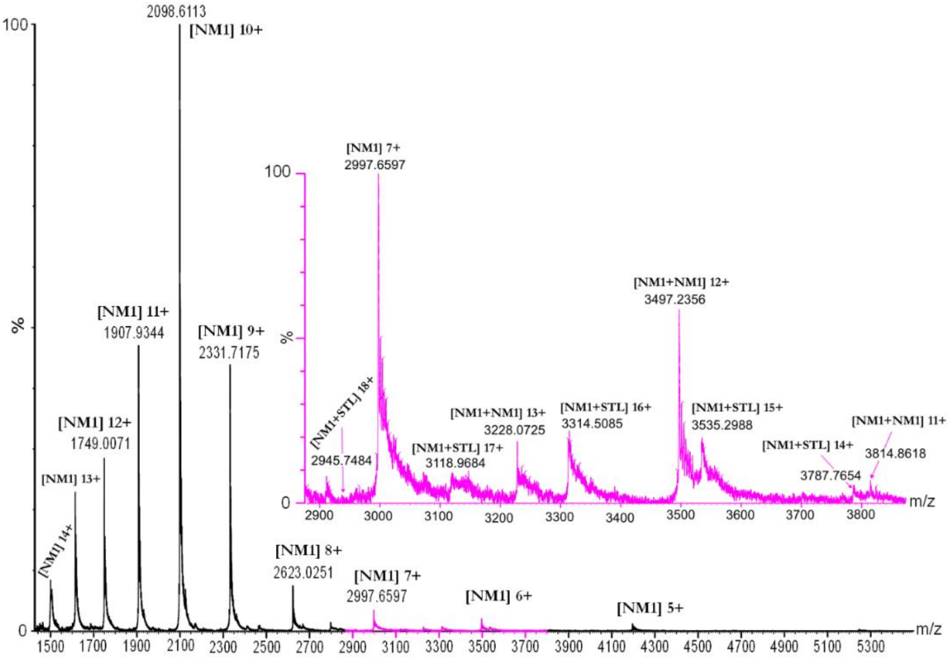


**(a)**

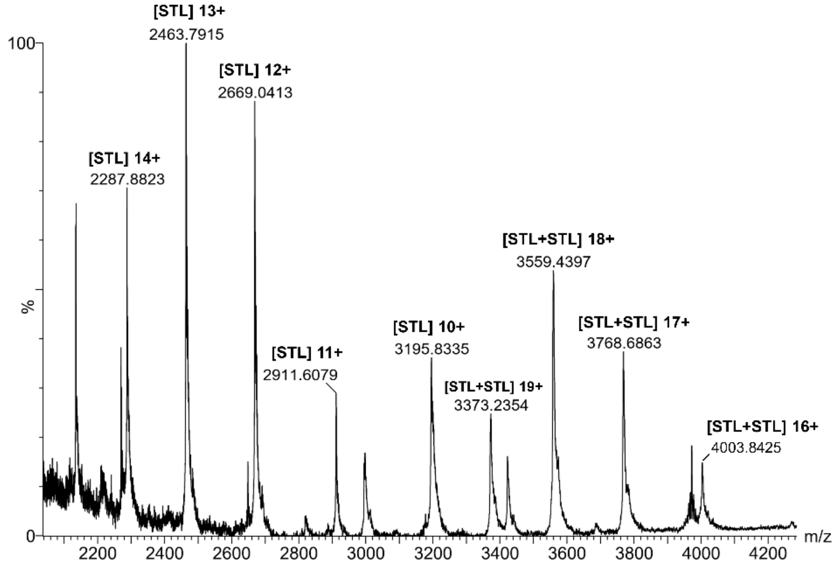


**(b)**

**Figure S1.** Annotated mass spectra of the mixture of the DUTφNM1 (NM1) with Stl (STL) protein **(a)** and Stl protein **(b)** measured under native electrospray conditions. **(a)** Main panel annotates the series of peaks corresponding to the DUTφNM1 protein. In addition, peaks corresponding to DUTφNM1 dimer and those of the complex of Stl with DUTφNM1 could also be detected in the spectra, as shown in the inset. In the inset the 2900-3900 m/z region of the spectra is magnified (this segment is shown in violet in the main panel, as well). Inset highlights the peaks corresponding to the heteromer with 53 kDa molar mass, which indicates the presence of a 1:1 Stl - DUTφNM1-Stl complex, corresponding to the added molar mass of one DUTφNM1 monomer (21 kDa) and one Stl monomer (32 kDa) (cf. also Figure 2d of the main text). (**b**). Peaks corresponding to Stl dimer (marked as STL+STL) (64 kDa) and monomer (marked as STL) (32 kDa) are annotated.

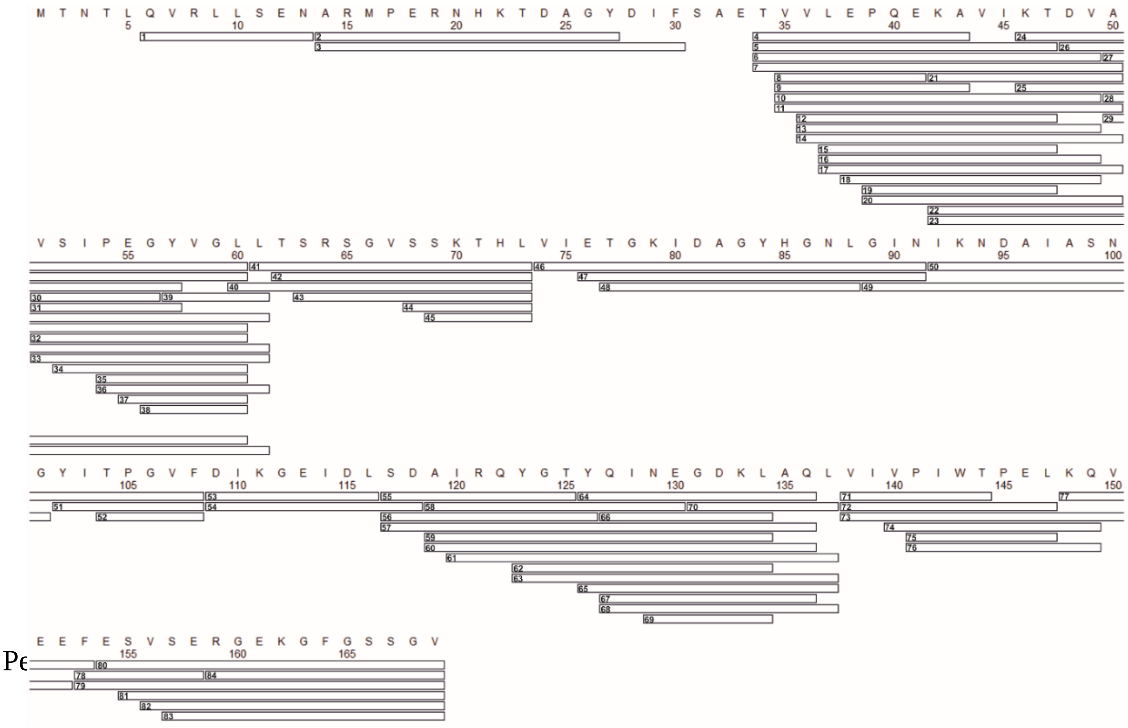


(**a**)

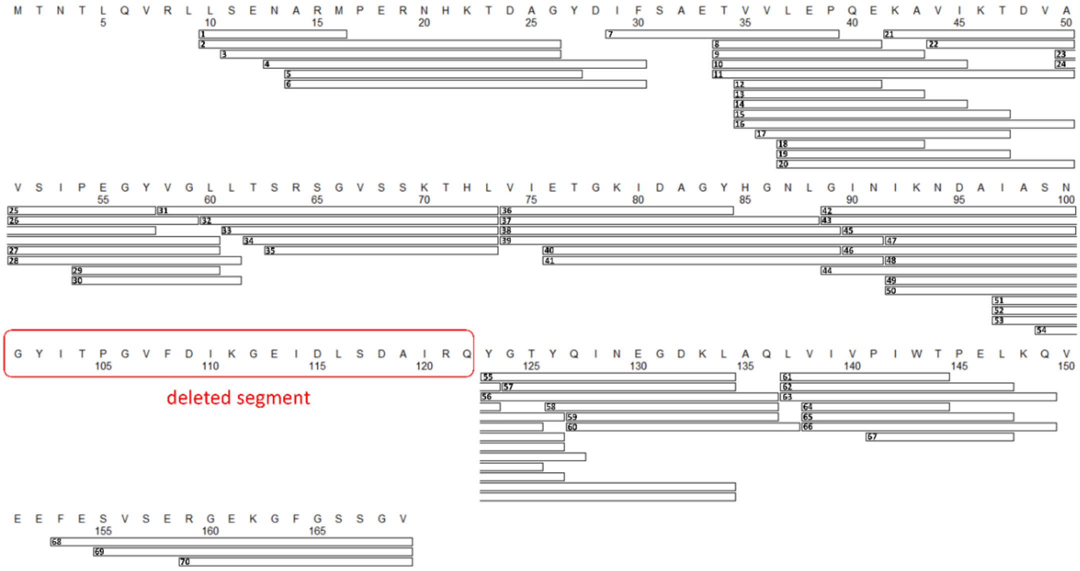


(**b**)

**Figure S2. Coverage map of HDX-MS difference plots for DUTφ11 and DUTφ11$^{\Delta insert}$ upon complex formation with Stl**. (**a**) Coverage map describing the distribution of 84 individual peptides (horizontal bars) covering 95.3% of the DUTφ11 sequence with redundancy of 6.0 in the presence of Stl. (**b**) Coverage map describing the distribution of 70 individual peptides (horizontal bars) covering 92% of the DUTφ11$^{\Delta insert}$ sequence with redundancy of 6.2 in the presence of Stl.

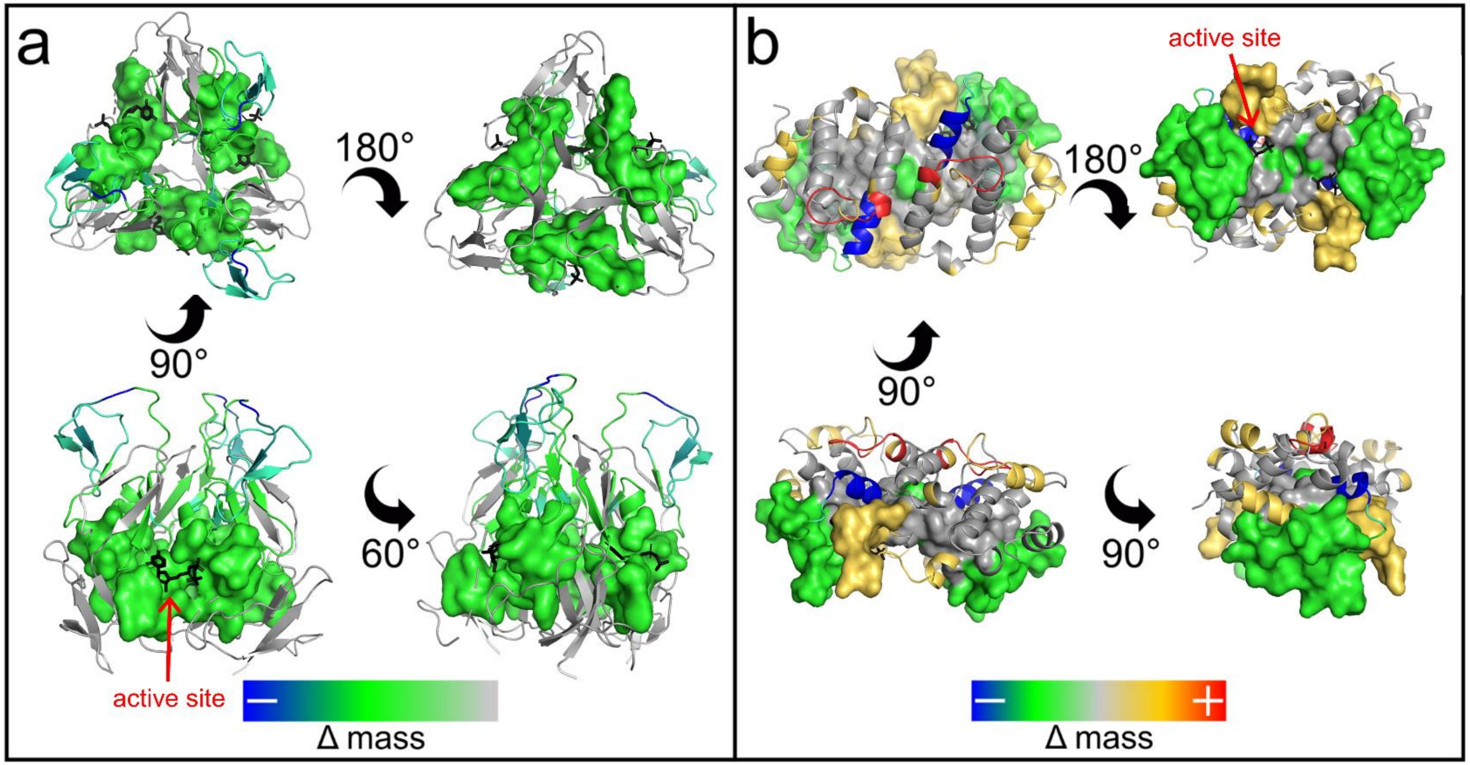


**Figure S3 Active sites of DUTφ11 and DUTφNM1 colored according to HDX-MS difference data obtained upon complex formation of those with Stl protein.** (**a**) X-ray crystal structure (PDB ID 4GV8 [1]) of DUTφ11 colored according to HDX-MS difference data following the color gradient shown at the bottom of the panel, surface representation is applied for active site residues (cf. boxed residues on Figure 3c of the main text) while other parts of the protein are shown as cartoon. Substrate analogue dUPNPP is also shown as black sticks in order to ease visualization of the active sites. (Views: top, bottom, sides.) (**b**) Homology model of DUTφNM1 colored according to HDX-MS difference data following the color gradient shown at the bottom of the panel, surface representation is applied for active site residues (cf. boxed residues on Figure 4c of the main text) while other parts of the protein are shown as cartoon. Substrate analogue dUPNPP is also shown as black sticks in order to ease visualization of the active sites. (Views: top, bottom, sides.)

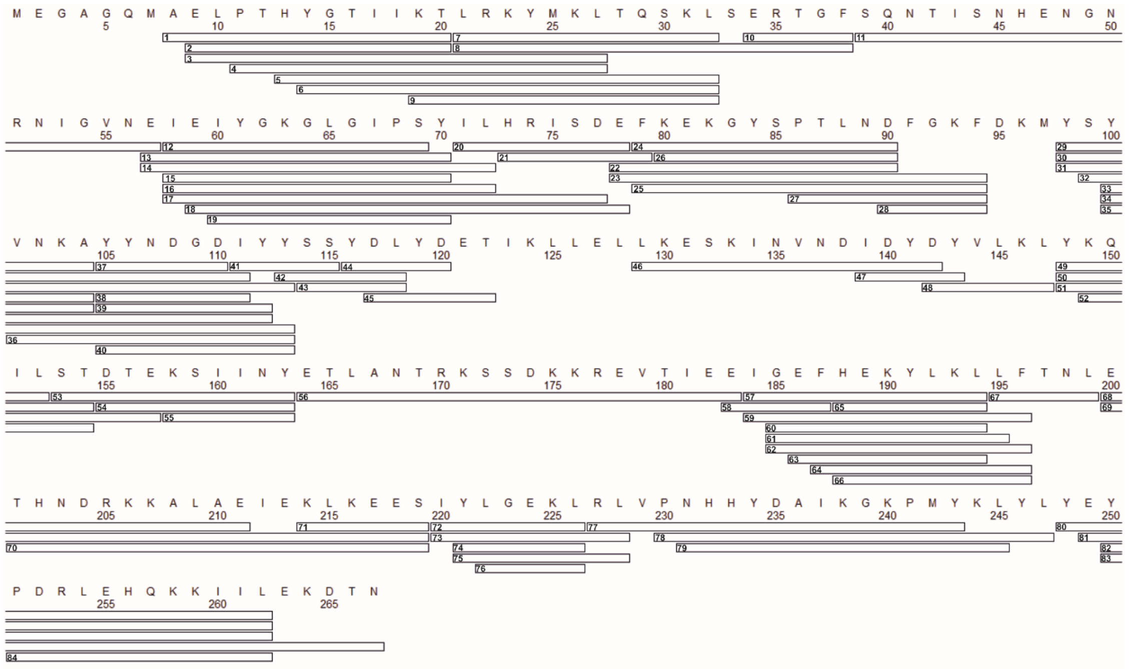


(**a**)

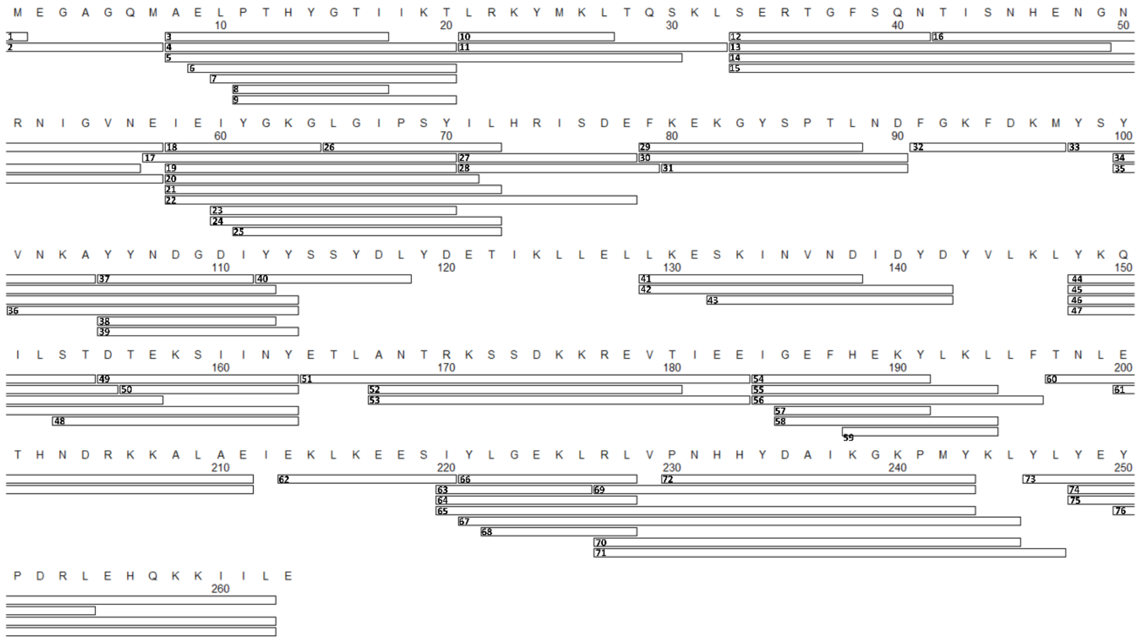


(**b**)

**Figure S4. Coverage map of HDX-MS difference plots for Stl upon complex formation with DUTφ11 and DUTφ11$^{\Delta insert}$.** (**a**) Coverage map describing the distribution of 84 individual peptides (horizontal bars) covering 94.0% of the Stl sequence with redundancy of 3.8 in the presence of DUTφ11. (**b**) Coverage map describing the distribution of 76 individual peptides (horizontal bars) covering 93.7% of the Stl sequence with redundancy of 3.7 in the presence of DUTφ11$^{\Delta insert}$.

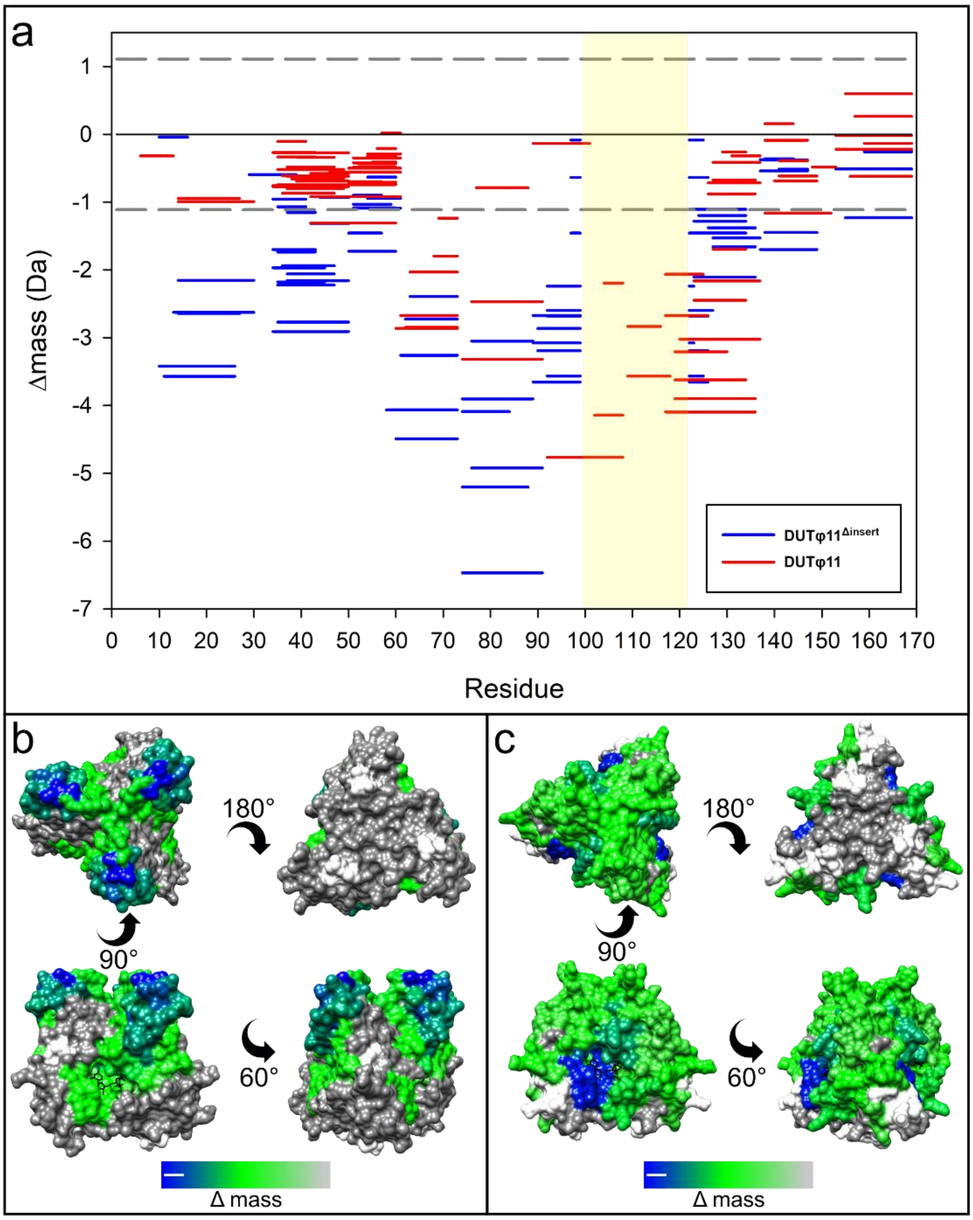


**Figure S5. HDX-MS difference data obtained for DUTφ11 and DUTφ11$^{\Delta insert}$ upon complex formation with Stl.** (**a**) Difference plots of DUTφ11 and DUTφ11$^{\Delta insert}$ upon complex formation with Stl. HDX difference plots the mass change (Δmass) of each individual peptide of dUTPases upon mixing with Stl. The dashed line represents the 99% confidence bands evaluated over the whole dataset. Numbering of residues in DUTφ11$^{\Delta insert}$ follow that of DUTφ11, yellow background highlights the deleted segment. Representation of the HDX-MS difference data on the surface of the DUTφ11 (**b**) and DUTφ11$^{\Delta insert}$ (**c**). In case of DUTφ11 the experimentally determined structure is shown (PDB ID: 4GV8), note that the position of the C-terminal 15 residues was not resolved in the crystal structure possibly due to flexibility. A three-dimensional structural model generated by SWISS-MODEL [2] is displayed for the truncated mutant. The substrate analogue is shown as black sticks in order to ease visualization of the interference of Stl and substrate binding. The coloring is according to the scale at the bottom of the panels. (Views: sides, top, bottom.) Regions which could not be probed by HDX-MS are shown in white.

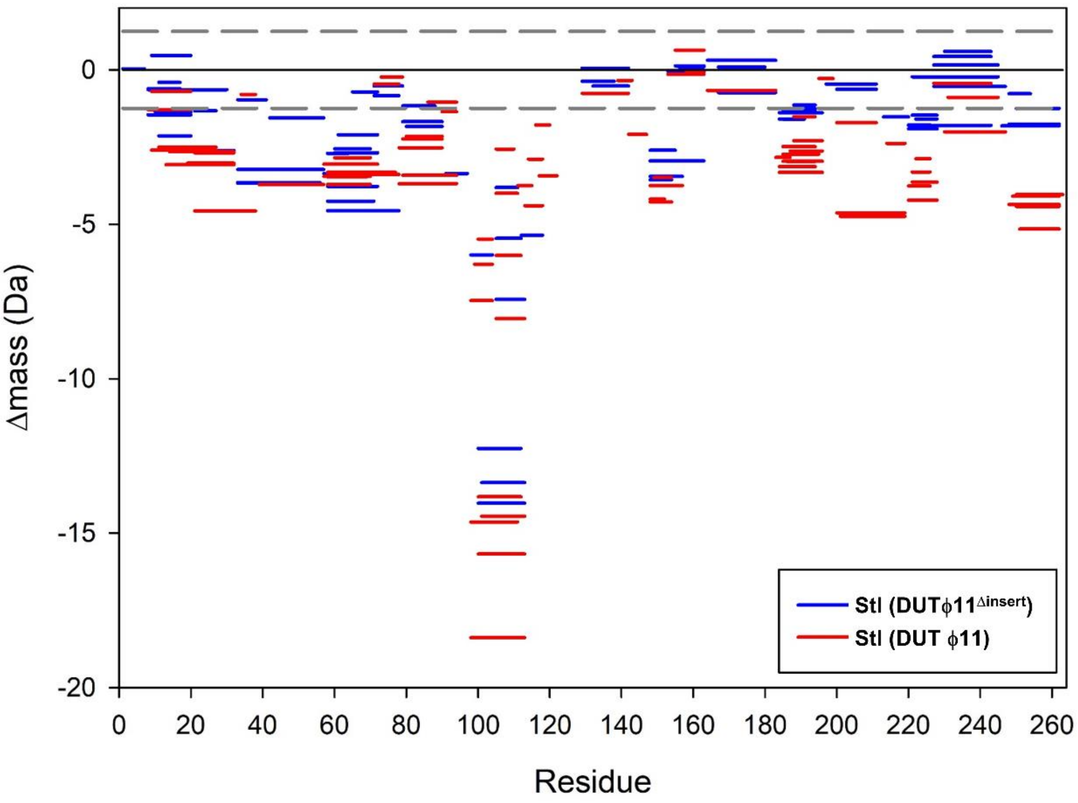


**Figure S6. HDX-MS difference data obtained for Stl upon complex formation with DUTφ11 and DUTφ11$^{\Delta insert}$.** HDX difference plots the mass change (Δmass) of each individual peptide of Stl upon mixing with DUTφ11 (red) and DUTφ11$^{\Delta insert}$ (blue). The dashed line represents the 95% confidence bands evaluated over the whole dataset.

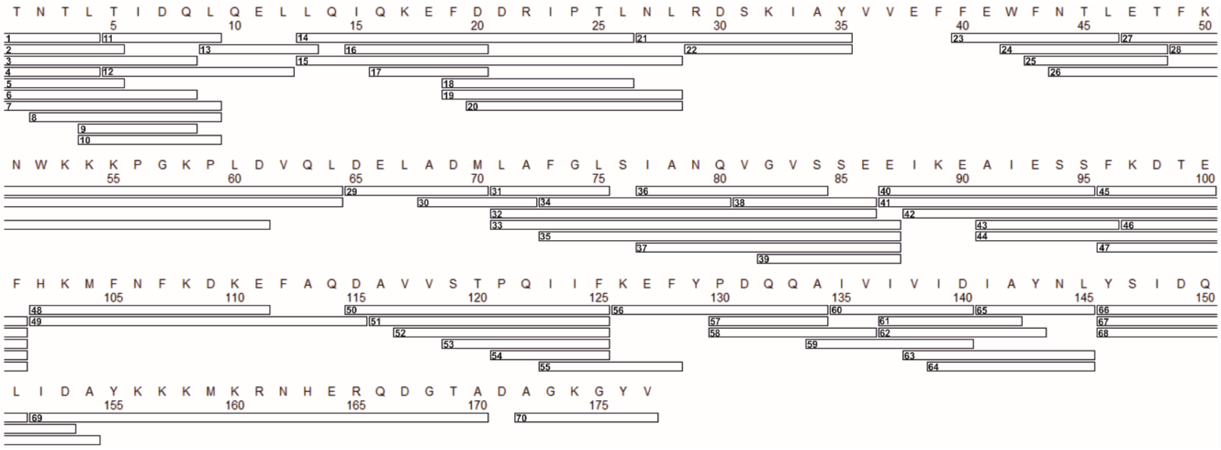


**Figure S7. Coverage map of HDX-MS difference plots for DUTφNM1 upon complex formation with Stl**. Coverage map describing the distribution of 70 individual peptides (horizontal bars) covering 97.3% of the DUTφNM1 with redundancy of 3.5 in the presence of Stl. (DUTφNM1 denotes residues 2-178 of φNM1 phage dUTPase, Uniprot ID: A0EWK2)

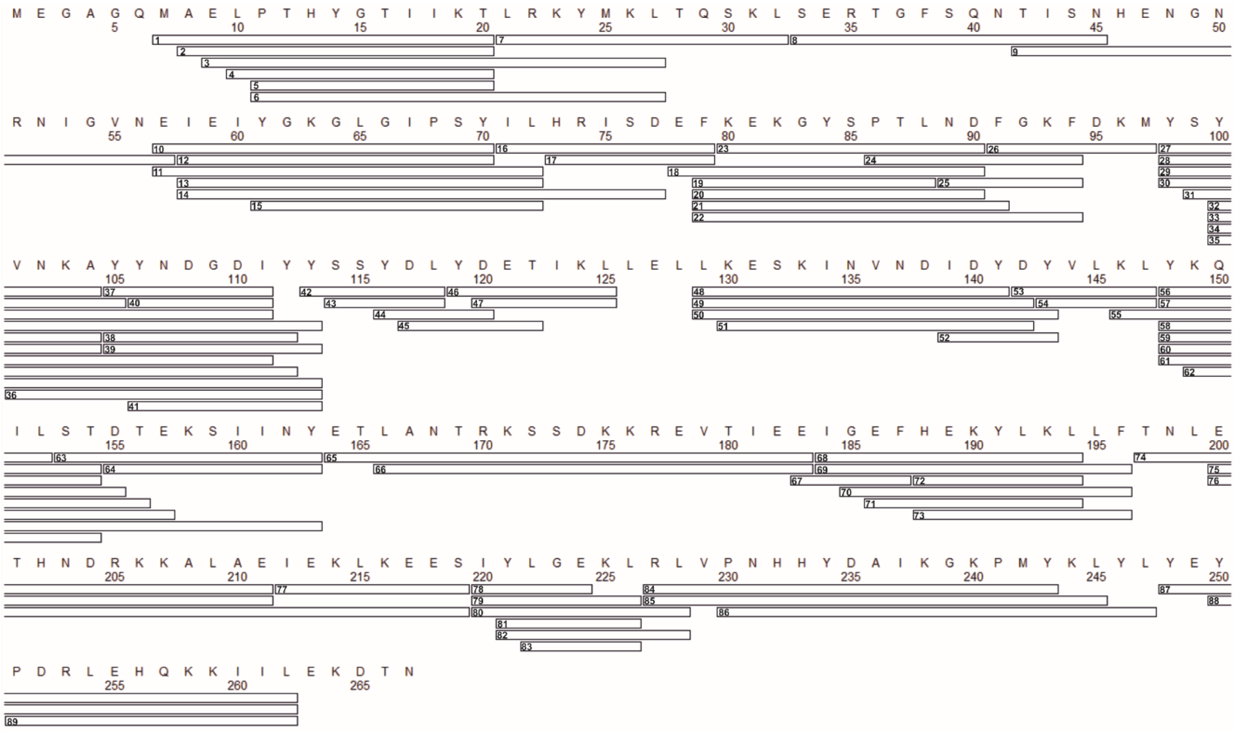


**Figure S8. Coverage map of HDX-MS difference plots for Stl upon complex formation with DUTφNM1.** Coverage map describing the distribution of 89 individual peptides (horizontal bars) covering 94.8% of the Stl sequence with redundancy of 3.8 in the presence of DUTφNM1.

```
ϕ11         MTNTLQVRLLSEN--------ARMPERNHKTD------AGYDIFSAETVVLEPQEKAVIK
ϕNM1        MTNTLTIDQLQELLQIQKEFDDRIPTLNLRDSKIAYVVEFFEWFNTLETFKNWKKKPGKP
            ***** :  *.*          *:*  * : .        :: *.:  .. : ::*.

ϕ11         TDVAVSIPEGYVGL---LTSRSGVSS-KTHLVIETGKIDAGYHGNLGIN---IKNDAIAS
ϕNM1        LDVQLDELADMLAFGLSIANQVGVSSEEIKEAIESSFKDTEFHKMFNFKDKEFAQDAVVS
             ** :.   . :.:   ::.: **** : : .**:.  *: :*  :.::   : :**:.*

ϕ11         NGYITPGVFDIKGEIDLSDAIRQYGTYQINEGDKLAQLVIVPIWTPELKQVEEFESVSER
ϕNM1        TPQIIFKEFYPDQQAIVIVIDIAYNLYSIDQ-------LID-AYKKKMKRNHERQDGTAD
               .  *    *  . :  :      *. *.*::       :*   :.  ::*: .* :. :

ϕ11         GEKGFGSSGV
ϕNM1        AGKGYV----
            . **:
```

**Figure S9. Sequence alignment of φ11 phage trimeric dUTPase and φNM1 phage dimeric dUTPase generated by NPS@ server** [3]**.** Conserved motifs are with bold. Identical residues denoted with stars (*) strongly similar residues denoted with colons (:), weakly similar residues denoted with dots (.). Identical peptide segments between the two dUTPases of different classes are underlined (cf. also [4]).

## Supplementary Tables

**Table S1. Sequences of protein constructs used.** Residues in blue belong to the linkers and tags used in the cloning and expression / purification of the proteins. Numbering of the first residue of a particular construct is indicated by the number in brackets and relates to the biologically occurring protein (encoded in Staphylococcus and its phages). In the case of the insert-deleted mutant (DUTφ11Δinsert ), the same first residue is indicated as for the wild type protein.

| Name | Molar extinction coefficient at 280 nm ($M^{-1}$ $cm^{-1}$) | Sequence |
|---|---|---|
| DUTφNM1 | 19940 | GSMAS [2] TNTLTIDQLQELLQIQKEFDDRIPTLNLRDSKIAY VVEFFEWFNTLETFKNWKKKPGKPLDVQLDELADMLAFGLSIA NQVGVSSEEIKEAIESSFKDTEFHKMFNFKDKEFAQDAVVSTP QIIFKEFYPDQQAIVIVIDIAYNLYSIDQLIDAYKKKMKRNHE RQDGTADAGKGYV |
| DUTφ11 | 14440 | MGSSHHHHHHSSGLVPRGSH [1] MTNTLQVRLLSENARMPERN HKTDAGYDIFSAETVVLEPQEKAVIKTDVAVSIPEGYVGLLTS RSGVSSKTHLVIETGKIDAGYHGNLGINIKNDAIASNGYITPG VFDIKGEIDLSDAIRQYGTYQINEGDKLAQLVIVPIWTPELKQ VEEFESVSERGEKGFGSSGV |
| DUTφ11$^{\Delta insert}$ | 12950 | [1] MTNTLQVRLLSENARMPERNHKTDAGYDIFSAETVVLEPQ EKAVIKTDVAVSIPEGYVGLLTSRSGVSSKTHLVIETGKIDAG YHGNLGINIKNDAIASNYGTYQINEGDKLAQLVIVPIWTPELK QVEEFESVSERGEKGFGSSGV |
| Stl SaPIbov1 | 35760 | GSPEFS [1] MEGAGQMAELPTHYGTIIKTLRKYMKLTQSKLSER TGFSQNTISNHENGNRNIGVNEIEIYGKGLGIPSYILHRISDEF KEKGYSPTLNDFGKFDKMYSYVNKAYYNDGDIYYSSYDLYDETI KLLELLKESKINVNDIDYDYVLKLYKQILSTDTEKSIINYETLA NTRKSSDKKREVTIEEIGEFHEKYLKLLFTNLETHNDRKKALAE IEKLKEESIYLGEKLRLVPNHHYDAIKGKPMYKLYLYEYPDRLE HQKKIILEKDTN |

## Supplemental References


1. Leveles, I.; Németh, V.; Szabó, J.E.; Harmat, V.; Nyíri, K.; Bendes, A.A.; Papp-Kádár, V.; Zagyva, I.; Róna, G.; Ozohanics, O.; et al. Structure and enzymatic mechanism of a moonlighting dUTPase. *Acta Crystallogr. Sect. D Biol. Crystallogr.* **2013**, *69*, 2298–308.
2. Biasini, M.; Bienert, S.; Waterhouse, A.; Arnold, K.; Studer, G.; Schmidt, T.; Kiefer, F.; Cassarino, T.G.; Bertoni, M.; Bordoli, L.; et al. SWISS-MODEL: modelling protein tertiary and quaternary structure using evolutionary information. *Nucleic Acids Res.* **2014**, *42*, W252–W258.
3. Combet, C.; Blanchet, C.; Geourjon, C.; Deléage, G. NPS@: network protein sequence analysis. *Trends Biochem. Sci.* **2000**, *25*, 147–50.
4. Hill, R.L.L.; Vlach, J.; Parker, L.K.; Christie, G.E.; Saad, J.S.; Dokland, T. Derepression of SaPIbov1 Is Independent of φNM1 Type 2 dUTPase Activity and Is Inhibited by dUTP and dUMP. *J. Mol. Biol.* **2017**, *429*, 1570–1580.


© 2019 by the authors. Submitted for possible open access publication under the terms and conditions of the Creative Commons Attribution (CC BY) license (http://creativecommons.org/licenses/by/4.0/).
